# Characterization of an industry-grade CMOS camera well suited for single molecule localization microscopy – high performance super-resolution at low cost

**DOI:** 10.1038/s41598-017-14762-6

**Published:** 2017-10-31

**Authors:** Robin Diekmann, Katharina Till, Marcel Müller, Matthias Simonis, Mark Schüttpelz, Thomas Huser

**Affiliations:** 1 Department of Physics, Bielefeld University, Bielefeld, Germany; 2 Micron Oxford, Department of Biochemistry, University of Oxford, Oxford, UK

## Abstract

Many commercial as well as custom-built fluorescence microscopes use scientific-grade cameras that represent a substantial share of the instrument’s cost. This holds particularly true for super-resolution localization microscopy where high demands are placed especially on the detector with respect to sensitivity, noise, and also image acquisition speed. Here, we present and carefully characterize an industry-grade CMOS camera as a cost-efficient alternative to commonly used scientific cameras. Direct experimental comparison of these two detector types shows widely similar performance for imaging by single molecule localization microscopy (SMLM). Furthermore, high image acquisition speeds are demonstrated for the CMOS detector by ultra-fast SMLM imaging.

## Introduction

The advance of multiple super-resolution fluorescence microscopy techniques^1^, such as single molecule localization microscopy (SMLM), structured illumination microscopy (SIM)^2,3^, stimulated emission depletion (STED)^4,5^, and super-resolution fluctuation imaging (SOFI)^6^ has had a huge impact on the life sciences within the last couple of years. The possibly highest spatial resolution among the above mentioned methods in combination with a relatively low technical complexity has contributed to the widespread dissemination of different SMLM approaches like (fluorescent) photo-activated localization microscopy ((F)PALM)^7,8^, (DNA-) point accumulation in nanoscale topography ((DNA-) PAINT)^9,10^, and (direct) stochastic optical reconstruction microscopy ((*d*)STORM)^11,12^. These make use of the temporal separation between individual emitters and overcome the diffraction limit by determining their position with nanometer precision and accuracy. As the fluorescent signal of single emitters has to be detected with sufficiently high signal to noise ratios (SNR), sensitive and low-noise photon detectors are a prerequisite for fluorescence detection. This was achieved by using electron multiplying charge-coupled device (EM-CCD) cameras in the first realizations of SMLM^7,11,12^. The advent of scientific-grade complementary metal-oxide-semiconductor (sCMOS) cameras resulted in a gradual replacement of the EM-CCD architecture in many SMLM implementations^13–19^. Prime movers of this trend are the usually lower cost and higher frame rates of sCMOS cameras. Furthermore, except for very low signal levels, higher localization precisions can be achieved^15^, which is one of the major performance marks in SMLM. This is complemented by the development of advanced fluorophores^20^, superior imaging buffer compositions^21^, as well as sophisticated approaches to tailoring the on-state time of fluorescent labels^22^. These lead to a substantial increase in the brightness of the probes and reduce the demands on the detector to some extent.

While Holm *et al*.^23^ have recently begun to demonstrate the use of a standard charge-coupled device (CCD) camera in a cost-efficient SMLM setup, the use of an industry-grade complementary metal-oxide-semiconductor (CMOS) camera for this purpose was also just demonstrated by Ma *et al*.^24^. These works have, however, focused on presenting cost efficient setups rather than extensively analyzing the individual components and carefully characterizing the camera performance for SMLM. In the latter publication, the CMOS camera was only theoretically compared to other camera architectures. One drawback of the Sony IMX265 CMOS sensor utilized there for the frequently preferred magnification of 60 × is the rather small pixel width of 3.45 µm. This leads to a suboptimal projected pixel width^25,26^ of 57.5 nm or a loss of 75% of the maximally possible field-of-view in case of 2 × 2 binning to achieve projected pixel widths of 115 nm. In contrast, we demonstrate the use of an industry-grade camera that uses the Sony IMX174LLJ-C image sensor, which features a pixel width of 5.86 µm, leading to projected pixel widths of 97.7 nm in case of 60× magnification. We characterize this CMOS camera in detail in this work. Focusing mainly on its utilization in SMLM, we furthermore systematically compare it experimentally and theoretically to a frequently used camera based on the sCMOS architecture^15^. While not making compromises regarding the other components of the microscope setup or experimental design, our findings reveal that both cameras show comparable performance for conventional *d*STORM imaging. Hence, the utilization of the industry-grade CMOS camera in standard setups can contribute to a considerable cost reduction in *d*STORM and other SMLM techniques without remarkably compromising the resulting images.

## Results

### Detector characterizations

The industry-grade IDS µeye UI-3060CP-M-GL Rev.2 CMOS camera features a detector size of 1936 × 1216 pixels. Using an appropriate magnification for SMLM (in our case approx. 53×) that is matched to the physical camera pixel widths of 5.86 µm results in projected pixel widths of about 110 nm. Accordingly, this would allow for imaging over a field-of-view (FOV) of approx. 213 µm × 134 µm. However, such a large FOV exceeds the illuminated area in most implementations for *d*STORM imaging, though it has recently been demonstrated how to adapt the illumination schemes in order to use the large detector areas of modern sCMOS cameras more efficiently^27–29^. All characterizations presented in this work were performed for a region of 512 × 512 pixels in the center of the chip, which corresponds to a FOV size of approx. 56 µm × 56 µm on our setup, and at a frame rate of 40 frames per second (fps). The camera was controlled using Micro Manager 1.4 (ref.^30^). In order to run it stably at high frame rates with this open source software package, we have adapted the IDS Micro Manger device adapter and make this modification available for download at ref.^31^ so that other users can easily benefit and use the same CMOS camera for their applications.

**Figure 1:**
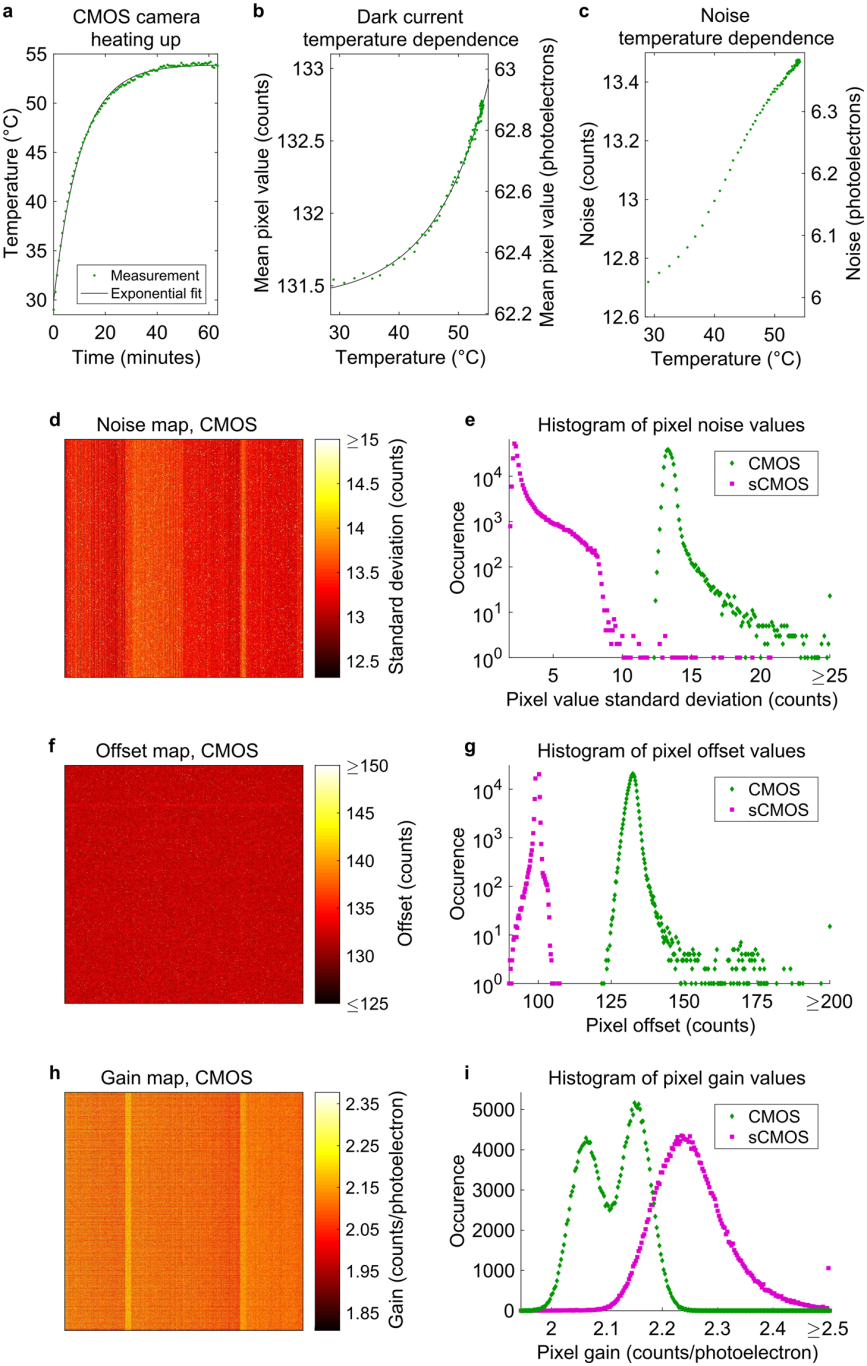
Camera characteristics. **(a)** The CMOS camera heats up to about 54 °C when operated at room temperature. (**b**,**c)** The mean dark current increases by about 1.3 ADU counts which corresponds to approx. 0.62 electrons during this phase of heating, while the mean noise rises by about 6% to 6.40 electrons. The single frame exposure times were 25 ms. (**d,e**) The noise pattern of a 512 × 512 pixels ROI reveals prominent vertical stripes for the CMOS camera, which shows remarkably higher noise than the sCMOS camera. (**f**,**g**) No obvious pattern is visible in the offset map of the CMOS camera, but the width of the distribution is notably higher than in comparison to the sCMOS camera. (**h**,**i**) The gain map of the CMOS camera shows similar stripes as the noise map and its distribution features two distinct peaks.

We found that the camera’s metal housing heats up and thermally equilibrates in about 52 minutes to approx. 54 °C when operated at room temperature (Fig. 1a). An exponential function fits well to the data which gives rise to the assumption that the temperature stabilizes at the asymptotic value of (53.9 ± 0.2) °C. It can be hypothesized that the camera chip reaches an even higher temperature than the housing. Hence, all measurements were conducted after allowing sufficient time for the camera to heat up. The mean pixel values of the dark detector follow an approximate exponential function of the temperature (Fig. 1b). However, the highest values lay only 1.3 analog-to-digital unit (ADU) counts above the lowest values, corresponding to approx. 0.62 electrons while the noise rises by about 6% from approx. 6.05 to 6.40 electrons after the camera has warmed up (Fig. 1c).

**Figure 2:**
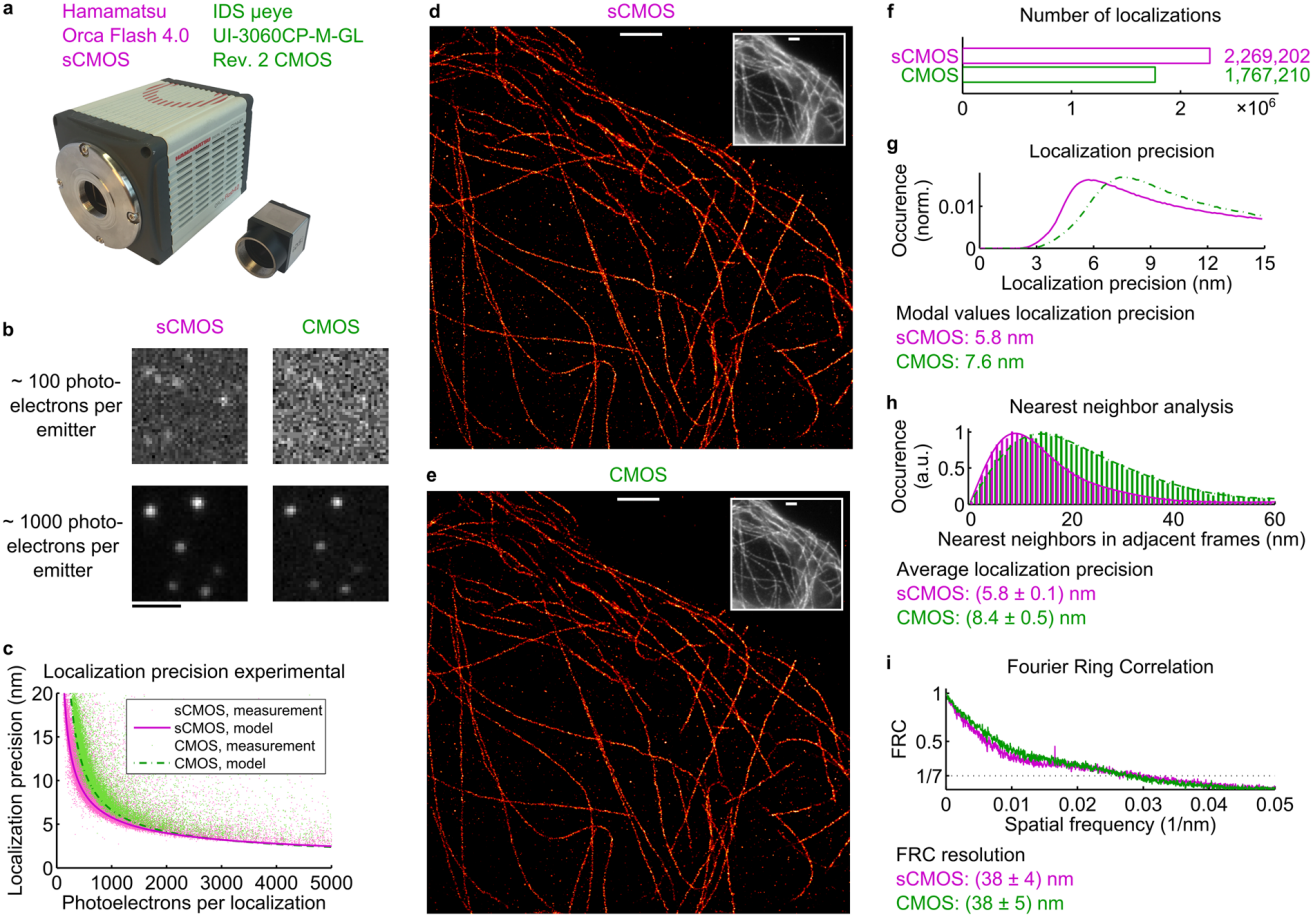
Direct experimental comparison between the industry-grade CMOS camera and an established scientific CMOS camera. (**a)** Photo of the Hamamatsu sCMOS camera and the IDS CMOS camera. (**b**) Simultaneous imaging of sub-diffraction sized fluorescent beads visualizes the difference in the signal-to-noise ratio at low photon counts which is less pronounced for higher signals. (**c**) Directly measured localization precisions for the two cameras diverge in particular for low photoelectron counts. (**d**,**e**) Simultaneous *d*STORM imaging of microtubules with both cameras exhibits no substantial difference in the reconstructed images. (**f**) Due to its lower quantum efficiency and SNR, fewer localizations are detected from the CMOS camera data in the *d*STORM experiment. Both the distributions of the localization precision estimated from the individual signal statistics (**g**) as well as the average value determined from a nearest neighbor analysis on the localizations table (**h**) reveal a slightly worse precision for the CMOS camera. Note that the localizations were filtered for values better than 15 nm. (**i**) The FRC resolution for both cameras is on the order of 38 nm, indicating that for this typical *d*STORM experiment the localization precision of the cameras is not the limiting parameter, but rather the spatial frequencies of the stained structure. Scale bars, 2 µm.

We have characterized the industry-grade IDS µeye UI-3060CP-M-GL Rev.2 CMOS camera as well as the scientific-grade Hamamatsu Orca Flash 4.0 sCMOS camera (Fig. 2a) chips by following the approach presented by Huang *et al*.^15^ (Supplementary Figure 1a). This resulted in pixel maps for noise, offset, and gain (Supplementary Note 1), i.e. the conversion factor from the number of detected photoelectrons to ADU counts by photon transfer curve measurements^32,33^. In principle, a homogeneous detector in terms of offset and gain is favorable for good performance in SMLM, and low noise values are beneficial. A pixelwise noise analysis (Fig. 1d,e) reveals that the majority of the pixels for the IDS µeye camera applied in the fluorescence measurements of this work (CMOS 1) shows a standard deviation in their dark current of 12.7 to 14.2 counts. Some pixels feature higher noise levels, which applies to 0.11% of all pixels. This statistics is confirmed by measuring the same parameter for two further cameras of the same type and manufacturer (CMOS 2 and CMOS 3) (Supplementary Figure 2). Of their pixels, also 0.11% show a standard deviation higher than 14.2 counts. The noise map (Fig. 1d) visualizes that the elevated pixel noise partially follows a pattern, as prominent stripes are visible. We have observed the same behavior also for other cameras of the same type (Supplementary Figure 2b,c). Pixels with elevated noise levels lead to a loss in localization precision in their direct environment^15,16^, so such a pattern supposedly leads to a spatially inhomogeneous localization precision. This prominent pattern does not show up in the pixelwise offset maps (Fig. 1f, Supplementary Figure 3), but the maps for the pixelwise gain (Fig. 1h, Supplementary Figure 4) also show vertically striped structures. As has been reported earlier^15,16^, the sCMOS camera shows remarkably less noise (Fig. 1e) and a narrower distribution of the pixel offset values (Fig. 1g). Both of these characteristics are favorable for better camera performance and these results are consistent with localization precision measurements (Fig. 2c,g,h). Furthermore, the sCMOS maps for noise, offset, and gain (Supplementary Figures 2, 3, and 4) show vertical stripe patterns in agreement with refs^15,33^. Huang *et al*.^15^ and Lin *et al*.^16^ have extensively discussed the use of sCMOS specific single emitter localization algorithms. This is possible by altering internal camera data-processing routines and more notably by explicitly taking the maps for pixel-dependent noise, offset and gain into account. However, many researchers successfully reconstruct sCMOS data with nonspecific, standard algorithms^17,22,28,34,35^ that can be chosen from a wide variety of software implementations^36^. Hence, we have utilized each camera as is, and have chosen to use the ThunderSTORM software^37^, the winner of 2013’s SMLM software benchmarking challenge^36^, because its underlying localization algorithm showed reliable performance when using its default settings which were not changed to compare the cameras. As explicit camera specific parameters, we have used the measured maps for offset and gain to set the reconstruction software settings to their mean values in each case.

### Performance in single molecule detection and localization

We used a custom-built inverted fluorescence microscope setup (Supplementary Figure 1b) to compare the performance between the sCMOS camera and the industry-grade CMOS camera (Fig. 2a) for single emitter localization. By equally dividing the fluorescence signal by a 50/50 beamsplitter cube to both cameras, samples were imaged simultaneously by both detectors at matched projected pixel sizes (Methods) to allow for a comparison that is as fair as possible.

Both, labeling density^38^ and sample drift^39^ have substantial influence on the spatial resolution of localization microscopy images but are not mainly affected by the detector. Localization precision^40^ also considerably determines the obtainable resolution but strongly depends on the specific camera that is utilized. To characterize this effect, we first recorded 30 stacks consisting of 1,000 frames each of immobilized, sub-diffraction sized 100 nm fluorescent beads at different illumination intensity levels (Fig. 2b). This allowed us to mimic the signal from single fluorophores at photon count rates comparable to raw localization microscopy data and running it through the single emitter detection software. These beads, however, emitted continuously for the entire 1,000 frames such that the localization precision^40^ could be measured directly for each bead as the standard deviation of the localized positions. Among other factors, the localization precision depends on Poisson-distributed photon shot noise and camera noise^25,26^. As the former is a function of the number of detected photons, the quantum efficiency is an important measure to compare the performance of different cameras. For the deep red spectral range at about 660 nm, the spectral range where a major share of *d*STORM experiments is conducted, the sCMOS camera features approx. 69% quantum efficiency^41^ while the CMOS camera features approx. 48% quantum efficiency^42^. Roughly estimated, the localization precision scales with the inverse of the square root of the number of detected photons per localization, such that a 20% better localization precision could be expected for the sCMOS camera when only taking these considerations into account. A more detailed evaluation based on the model presented by Mortensen *et al*.^26^ that incorporates additional factors predicts an even higher value depending on the number of photons that reach the detector, e.g. a 35% better precision in case of 5,000 photons and 83% better precision in case of 1,000 photons (Supplementary Figure 5). Note that these relations change for a different spectral range, e.g. the CMOS camera has a peak quantum efficiency of more than 75% at wavelengths around 500 nm while the sCMOS camera has a peak quantum efficiency of about 72% for wavelengths around 580 nm (Supplementary Figure 6).

The uncooled CMOS camera features increased camera noise as compared to the sCMOS camera (Fig. 1e) and therefore suffers from an inferior SNR. This becomes visible by the direct comparison between the two cameras at low signal strengths of about 100 detected photoelectrons per emitter (Fig. 2b). However, in case of about 1,000 detected photoelectrons per emitter, visual inspection of the data reveals more comparable SNRs due to the increased signal. Plotting the localization precision as a function of the number of detected photoelectrons per localization shows the influence of the SNR (Fig. 2c). At low numbers of photoelectrons per localization, the experimentally determined localization precision of the CMOS camera is notably worse than the localization precision of the sCMOS camera. E.g. the localization precision is about 9 nm for the sCMOS camera in case of 500 detected photoelectrons while it is about 12 nm for the CMOS camera, and, hence approx. 33% worse. However, the experimentally determined localization precisions converge for about 2,000 photoelectrons per localization or more. We utilize the model of Mortensen *et al*.^26^ to check for consistency of the experimentally determined localization precision. The theoretical prediction coincides with the high density regions of the data points at around 1,000 photoelectrons per localization, while the fit is better for the sCMOS camera. The worse experimental values in comparison to the prediction might be due to the less homogeneous detector in case of the CMOS camera (see e.g. the noise, offset and gain maps in Fig. 1d,f,h). At high signal levels of multiple thousand photoelectrons, the experimentally achieved localization precisions do not reach the theoretically predicted values for either camera, though. This might be due to uncorrected spatial drifts owing to thermal and/or mechanical instabilities of our setup. Additionally, some data points have noticeable higher values than the average experimentally determined as well as theoretically predicted localization precision which is possibly due to erroneously detected noise in the single emitter reconstruction algorithmic pipeline. On purpose, we did not optimize the algorithmic parameters of the reconstruction software but used the default settings to compare the two cameras as impartially as possible. Please note that while the prediction based on camera parameters only (Supplementary Figure 5) also considers the quantum efficiency (Supplementary Figure 6), Fig. 2c shows the localization precision as a function of the number of detected photoelectrons, i.e. the quantum efficiency is not considered. Further deviations between the curves can be attributed to different camera properties (Supplementary Figure 7).

### Comparative ***d***STORM imaging

The considerations presented so far apply to general single molecule detection-based techniques ranging from, e.g., particle tracking^43^ to imaging approaches such as (F)PALM, and (*d)*STORM. Next, we sought to conduct a comparison of the cameras’ performance in the specific case of *d*STORM imaging. Again, the signal was split to both cameras and raw data were collected simultaneously. In this manner, we imaged Alexa 647 immunostained microtubules in fixed U2OS cells. Using default parameters for the single emitter fitting algorithm and equal settings for post-processing of both data sets resulted in super-resolved reconstructions of extensive similarity for the two cameras (Fig. 2d,e). However, 28% more emitters were detected with the applied algorithmic parameters in case of the sCMOS camera (Fig. 2f) which is presumably due to its higher quantum efficiency and better SNR. Using the quantitative measure of localization precision, the CMOS camera again performs slightly worse than the sCMOS camera (Fig. 2g,h). Both, the modal value of the single emitter localization precision as estimated directly from the signal statistics^13,26,37^ (Fig. 2g) and the average localization precision estimated from the analysis of nearest neighboring localizations in subsequent frames^44^ (Fig. 2h), are about 6 nm for the sCMOS camera and 8 nm for the CMOS camera. It should also be noted that each camera only detected half of the available signal due to the 50/50 beamsplitter, lowering the overall possible localization precision by about a factor of √2. The spatial resolution obtained by Fourier Ring Correlation (FRC)^45,46^ analysis, however, does not show a considerable difference and is about 38 nm for both cameras (Fig. 2i). This suggests that the main factor limiting the achievable spatial resolution is the distribution of the spatial frequencies imposed by the stained sample structure, but not the localization precisions, which is also implied by the similar visual impression of the reconstructed images. Hence, both cameras perform in practical terms equally well for this typical *d*STORM imaging scenario.

### **High-speed*d*STORM imaging**

**Figure 3:**
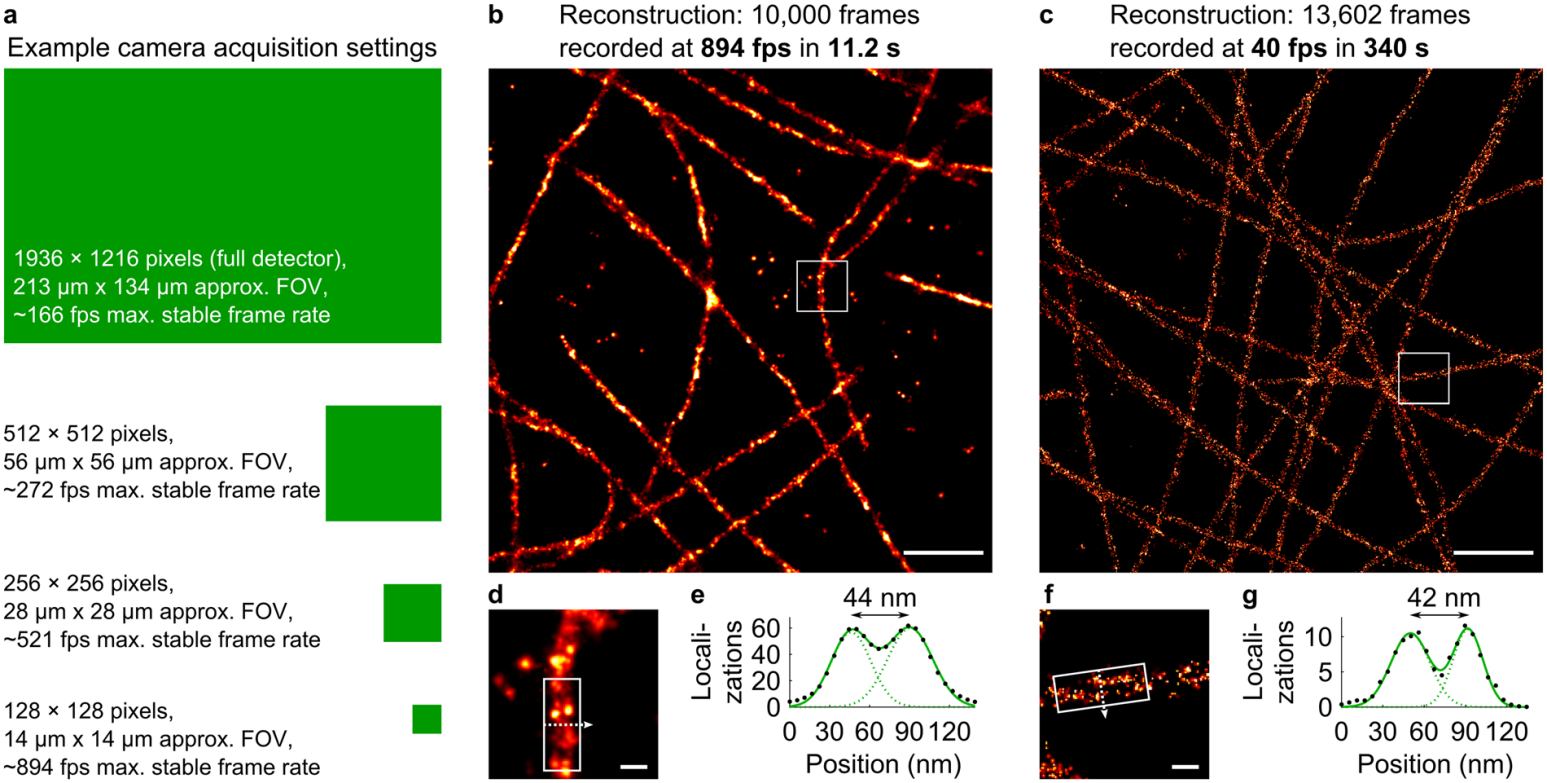
The CMOS camera allows for *d*STORM imaging over a wide range of frame rates and fields-of-view. **(a**) Dependent on the ROI, imaging with the CMOS camera can be performed at different maximum frame rates. The green boxes visualize the relations between the listed ROI dimensions. (**b**,**d)** A microtubule image reconstructed from 10,000 frames recorded at a rate of 894 fps. (**c**,**f**) Imaging a similar sample at 40 fps. (**e**,**g**) Line profiles along straight microtubules reveal the hollow microtubule structure both for high and low frame rate imaging. Scale bars on (**b**,**c)** 1 µm. Scale bars on (**d**,**f)** 100 nm.

A major advantage of (s)CMOS cameras over EM-CCD cameras is their usually much higher maximum imaging frame rate. Running the CMOS camera while reading out its full detector size of 1936 × 1216 pixels, we measured a maximum possible stable frame rate of approx. 166 fps (Fig. 3a) in agreement with the manufacturer specifications^42^. When decreasing the region of interest (ROI) size to 128 × 128 pixels, the image acquisition rate increased to approx. 894 fps. Using sufficiently high intensities of the laser for fluorescence excitation of 33 to 180 kW/cm² and additional UV activation, we were able to acquire *d*STORM images from 10,000 raw frames in 11.2 seconds (Fig. 3b), comparable to what has been demonstrated earlier by Lin *et al*. using an sCMOS camera^47^. The line profile (Fig. 3e) along an immunostained microtubule filament (Fig. 3d) shows its hollow structure^21,48,49^, thus indicating a resolution of better than 44 nm. By decreasing the frame rate to 40 fps while keeping the readout illumination constant but deactivating the UV activation (Fig. 3c,f,g), the localization precision was improved from about 9.2 nm to about 2.6 nm (Supplementary Figure 8), resolving the hollow structure of an immunostained microtubule filament even more clearly (Fig. 3g). As the localization precision is proportional to the theoretically achievable spatial resolution, this can also be enhanced by a factor of 3 to 4 when changing the imaging parameters. Hence, the CMOS camera enables to adjust the frame rate over a wide range that covers a large number of typical SMLM scenarios. Furthermore, the large detector size also permits the use of this camera in large FOV applications^27–29^ at sufficiently high frame rates.

## Discussion

We have evaluated and compared the established scientific-grade Hamamatsu Orca Flash 4.0 sCMOS camera and the industry-grade IDS µeye UI-3060CP-M-GL Rev.2 CMOS camera for their performance in super-resolution localization microscopy. While these specific models have been tested, both detectors are used in multiple commercially available camera models by different manufacturers. The presented approach of using the exact same methods for characterization and a custom setup that enables simultaneous imaging of the same sample by both cameras allows for a fair comparison of their overall performance. Hence, this method is generally applicable to run competitions between different detectors.

For this work, we have used the first generation of the Hamamatsu Orca Flash 4.0 sCMOS camera, while the third generation has recently become commercially available. Though some differences in their performance can be expected, the utilized first generation camera already shows superior performance when compared to the industry-grade CMOS camera particularly with respect to noise and quantum efficiency in the deep red spectral range. This leads to an experimentally confirmed higher localization precision for the sCMOS detector at low signal levels where relative differences are on the order of 33% at around 500 photoelectrons per localization but become less pronounced for more than 2,000 photoelectrons per localization. A few nanometers difference in the localization precision is not supposed to play a major role for many biological questions however. Consequently, *d*STORM imaging of biological samples showed no substantial difference in the FRC resolution. We therefore conclude that for *d*STORM scenarios such as imaging the popular dye Alexa 647, here demonstrated for microtubule labeling, one can resort to the tested CMOS camera without considerable drawbacks. As lower signal levels are more common in case of different SMLM scenarios such as (*d*)STORM with inferior dyes or (F)PALM, worse localization precision on the order of several nanometers might need to be accepted, but will still allow for imaging at resolutions well below the diffraction limit. Additionally, these probes often emit at shorter wavelengths where the CMOS camera features a quantum efficiency that is about 63% higher relative to the deep red spectral range, counteracting the effect of lower fluorescence signals. Optimizing the imaging conditions, the experimentally obtained localization precision is about 3 nm. This is easily on the order of label sizes (e.g. if antibodies are used) or residual drift effects even after correction, and, therefore, no significant difference for the considered camera types is expected.

We have used a custom setup with approx. 53× magnification for the CMOS camera, but the convenient pixel size of the presented CMOS detector also allows for its easy integration at projected pixel widths of 97.7 nm into microscopes with the frequently used 60× magnification. As it is connected via USB 3.0, it can simply be controlled by standard contemporary computers without the need for additional hardware. The reliable operation with the widely used free and open-source software Micro Manager is enabled by our open-source adaptation of the camera device adapter and has been successfully tested on multiple PCs. The camera size and weight are considerably smaller than that of commercially available sCMOS cameras which may make it a preferred choice in compact and mobile setups. The latter is also favored by the lack of moving parts, e.g. fans. The additional I/O connector can be used for camera triggering which has also been integrated by us into the camera device adapter. Hence, multiple cameras can easily be used in a synchronized manner, e.g. for multi-color imaging. The low cost might make it economically reasonable to integrate a second camera instead of a commercial image splitter.

While we have focused on the characterization for its use in SMLM, the CMOS camera properties also promise a highly efficient use in other approaches to high- and super-resolution microscopy. Certainly, novel industry-grade CMOS detectors with even superior quality will become available in the future. For instance, the Sony IMX250 CMOS sensor has substantially less camera noise and will enable a larger FOV when using the same projected pixel sizes. However, it features lower peak quantum efficiency, a suboptimal pixel width and a decreased frame rate for full chip readout. Nevertheless, properties of industry-grade CMOS cameras will presumably further approach the fundamental limits toward which scientific-grade cameras are already converging against today. Hence, the differences for new detector generations will probably become even fewer. In any case, our results show that even with the tested cameras and depending on the scenario, differences in the performance can be vanishing and may not be relevant for biological applications. We believe that this work can contribute to the current trend of cost reduction for instrumentation in optical nanoscopy. This will enable an even wider spread of super-resolution microscopes and further promote their role in answering fundamental questions that the life sciences are facing.

## Materials and Methods

### Camera setups

CMOS µeye cameras (UI-3060CP-M-GL Rev.2, IDS) were connected via USB 3.0 to Windows 7 PCs (PC hardware for *d*STORM measurements: Dell Latitude E5540, Intel i3-4030U CPU, 8GB memory, 64 bit OS) and run using the free open-source software Micro Manager version 1.4 (ref.^30^). The original device adapter software, providing the connection between the camera and Micro Manager, was extended to allow for high acquisition speeds. To this end, a buffered burst acquisition mode offered by the camera API (but so far not supported in MicroManager) was implemented, which allows for concurrent image acquisition and data transfer between camera and the control computer. The software was also extended to support switching of the camera’s GPIO pins for external triggering and control of light sources. The sCMOS camera was run using Micro Manager version 1.4 on a Windows 7 Desktop PC (Intel Xeon CPU W3680, 24 GB memory, 64 bit OS) with the default device adapter included with Micro Manager.

### Camera characterization

The camera chip characterization was performed following the approach of Huang *et al*.^15^ for a ROI of 512 × 512 pixels. For dark pixel offset and read noise measurements, it was ensured that the chip was in darkness during the acquisition of 4,000 to 8,192 frames. From this data, the baseline for each pixel was determined by the mean value and the read noise by the standard deviation over all frames. A sequence of 15 to 20 (CMOS 1: 15, CMOS 2: 16, CMOS 3: 17, sCMOS: 20) similar measurements with illumination on the chip at different light levels (Supplementary Figure 1a) up to approx. 1000 counts per pixel and per 25 ms exposure time allowed to determine the gain factor for each pixel. This was calculated via linear regression on the variance as a function of the mean signal (Supplementary Note 1). For temperature dependent baseline and noise measurements, the temperature of the camera metal housing was measured in intervals of 40 s and the mean pixel values as well as noise were calculated from 200 frames taken in this period.

### Optical setup

Fluorescence measurements were conducted on a custom-built SMLM setup. A 40 mW laser with 639 nm vacuum wavelength (Coherent) for fluorescence excitation was optionally expanded by a telescope (f = −30 mm and f = 160 mm, Qioptiq). The beam was filtered through an excitation filter (639DF9, Omega Optics) before focusing by a f = 200 mm lens (Qioptiq) to the back focal plane of the microscope objective lens (60× NA1.49 ApoN, Olympus). Before transmission through the focusing lens, the fluorescence excitation beam was combined with the beam from a 50 mW laser with 405 nm vacuum wavelength (OBIS, Coherent) for photoswitching. Mounting on a translation stage allowed for shifting the beam position at the back-focal-plane of the objective lens to switch from epi to TIRF illumination. A dichroic mirror (F63-T01, AHF) was used for spectral separation between excitation and emission. The fluorescence emission was additionally filtered (Razor Edge Long Pass 647, Semrock, and HQ 685/70, Chroma) and focused onto the camera chips. In case of the CMOS camera, we used a tube lens of f = 160 mm focal length (Qioptiq), resulting in measured backprojected pixel widths and heights of 109.7 nm. For direct comparison measurements to the sCMOS camera, the fluorescence emission beam was split by a 50/50 beamsplitter cube (G335520000, Qioptiq) to both cameras. In case of the sCMOS camera, we used a tube lens with f = 180 mm (Qioptiq) to achieve comparable values for the backprojected pixel widths and heights which we measured as 107.5 nm. The tube lenses were aligned by placing the f = 160 mm lens such that spherical aberrations were minimized in the signal for the CMOS camera. Afterward, the f = 180 mm lens was positioned such that both cameras shared the same focal plane on the sample.

### Bead sample preparation

Sub-diffration sized fluorescent Tetraspeck beads of 100 nm (ThermoFisher Scientific) were diluted at 1:40,000 from the stock into phosphate buffered saline (PBS) (Sigma Aldrich) and dried at room temperature (RT) in a Nunc Lab-Tek II chambered #1.5 coverslip (ThermoFisher Scientific). Before imaging, the chamber was filled with double-distilled water (ddH_2_O).

### Cell preparation

Human bone osteosarcoma (U2OS) cells were grown in Dulbecco’s Modified Eagle Medium (DMEM) (Sigma Aldrich) supplemented with 10% fetal bovine serum (FBS). A humidified CO_2_ atmosphere at 37 °C was used for cultivation on Nunc Lab-Tek II chambered #1.5 coverslips for about 24 hours before fixation. For fixation, the medium was aspirated and the cells were fixed for 20 minutes in 0.5% glutaraldehyde (Sigma Aldrich) in cytoskeleton stabilizing buffer pre-warmed to 37 °C that consisted of 80 mM PIPES (Sigma Aldrich), 1 mM Magnesium Chloride (Roth), 5 mM Ethylenediaminetetraacetic acid (EDTA) (Sigma Aldrich) in ddH_2_O with pH adjusted to 6.9 using an aqueous solution of potassium hydroxide (KOH) (Roth). Cells were washed 3 times with PBS and permeabilized with 0.1% Triton X-100 (Fluka) in PBS for 10 minutes at RT. After fixation and permeabilization, cells were washed three times with PBS and autofluorescence was quenched by incubating for 7 minutes with 0.2% w/v sodium borohydride (NaBH_4_) (Sigma Aldrich) in ddH_2_O, washed three times with PBS, incubated for 7 minutes with 50 mM Tris-HCl pH 8 (Roth) in ddH_2_O, and washed again three times with PBS. Samples were blocked with 5% w/v bovine serum albumin (BSA) (Sigma Aldrich) in PBS for 45 minutes. Immunostaining was done with primary antibodies against alpha-tubulin raised in mouse (Mouse anti-alpha tubulin Alexa Fluor 488 (B-5-1-2), Invitrogen) diluted 1:200 from the stock in PBS supplemented with 1% BSA, and 0.025% Triton X-100 for approx. 100 minutes. After washing three times with PBS, samples were incubated with Alexa 647-labeled secondary antibodies against mouse raised in goat (Alexa Fluor 647 F(ab’) 2 fragment of goat anti-mouse IgG (H+L), Invitrogen) diluted 1:200 from the stock in PBS supplemented with 1% BSA, and 0.025% Triton X-100 for approx. 100 minutes and subsequently excessively washed with PBS. The cells were additionally post-fixed with 1% v/v formaldehyde (ThermoFisher Scientific) in PBS for 5 minutes and again washed with PBS. Prepared cells were stored in PBS at 4 °C until imaging.

### Imaging buffer

*d*STORM buffer consisted of a glucose oxidase/catalase-based oxygen scavenger system supplemented with a thiol in a buffer system. An enzyme stock solution (ES) was prepared at 0.1 kU/mL glucose oxidase (Sigma Aldrich), 1.2 kU/mL catalase (Sigma Aldrich), 4 mM tris(2-carboxyethyl)phosphine (TCEP) (Sigma Aldrich), 25 mM potassium chloride (KCl) (Acros Organics), 20 mM Tris-HCl pH 7.5, and 50% v/v glycerol (Riedel-de Haen) in ddH_2_O. A glucose stock solution (GS) was prepared at 10% w/v glucose (Sigma Aldrich), and 10% glycerol in ddH_2_O. ES and GS were stored in aliquots at −20 °C for up to 4 months. We prepared the imaging buffer by mixing 480 µL GS, 520 µL ddH_2_O, 60 µL 1 M Tris-HCl pH 8, 60 µL 1 M NaCl (Sigma Aldrich), 12 µL 200 mM cyclooctatetraene (COT) (Sigma Aldrich) in dimethyl sulfoxide (DMSO) (Sigma Aldrich), 60 µL ES, and 12 µL 100% ß-mercaptoethanol (BME) (Sigma Aldrich) (preparation for measurement shown in Fig. 3b) or 8.4 µL BME (preparation for measurement shown in Figs 2d,e and 3c). Hence, the thiol concentrations in the imaging buffer were either 143 mM or 100 mM BME.

### Image acquisition and analysis

*d*STORM images were acquired at 4.5 to 6.3 kW/cm² (measurements shown in Fig. 2d,e) or 33 to 180 kW/cm² (measurements shown in Fig. 3b,c) illumination intensity using the 639 nm laser for fluorescence excitation in TIRF mode. For the measurements shown in Figs 2d,e and 3b, we additionally used the 405 nm laser for photoactivation in TIRF mode while the intensity was manually adjusted for each measurement but peak intensities did not exceed 0.02 kW/cm². Diffraction limited images were acquired prior to *d*STORM acquisition. Raw frames were recorded at rates of 40 fps (measurements shown in Figs 2d,e and 3c) or 894 fps (measurement shown in Fig. 3b). The data was recorded using Micro Manager 1.4 (ref.^30^), imported into Fiji^50^ and *d*STORM reconstructions were run using ThunderSTORM^37^ on its default settings. The localizations were filtered and the super-resolved reconstruction was rendered at pixel sizes of approx. 5.5 nm width and height using the normalized Gaussian option, i.e. each localization was smoothed individually with a Gaussian function of which the standard deviation corresponded to the localization precision as estimated by ThunderSTORM based on the signal statistics. For the result shown in Fig. 2d,e, we used the same post-processing steps of filtering for localization precision values less than 15 nm and correcting the drift via the build in cross-correlation function on substacks^39^ with 5 temporal bins. For the result shown in Fig. 3c, we set the initial point spread function sigma value to 1.2 pixels. Postprocessing consisted of filtering for localization precision values less than 10 nm, correcting drift as described above with 4 temporal bins, filtering for localizations that featured at least 100 neighbors within a spatial radius of 40 nm and merging of localizations in subsequent frames that lay within a spatial radius of 20 nm while a temporal delay of 1 frame was allowed. For the result shown in Fig. 3b, we also set the initial point spread function sigma value to 1.2 pixels. Postprocessing consisted of filtering for localization precision values less than 15 nm, filtering for localizations that featured at least 100 neighbors within a spatial radius of 100 nm and correcting drift as described above with 4 temporal bins. Image analysis was carried out using basic Fiji functions, custom written Matlab (Mathworks) scripts, and the FRC plugin^46^ for Fiji.

### Data availability

The data that support the images and plots within this paper and other findings of this study are available from the corresponding author upon reasonable request.

## Electronic supplementary material

Supplementary Information
