# Supplementary material for: Characterization of an industry-grade CMOS camera well suited for single molecule localization microscopy – high performance super-resolution at low cost
Source: Sci Rep. 2017 Oct 31;7:14425. doi: 10.1038/s41598-017-14762-6 (PMC5663701; doi:10.1038/s41598-017-14762-6)
Supplement: Supplementary file 1 — Supplementary Information [file 41598_2017_14762_MOESM1_ESM.pdf]

Supplementary Information

Characterization of an industry-grade CMOS camera well suited for single molecule localization microscopy – high performance super-resolution at low cost

Robin Diekmann[1], Katharina Till[1], Marcel Müller[1,2], Matthias Simonis[1], Mark Schüttpelz[1], Thomas Huser[1]

[1] Department of Physics, Bielefeld University, Bielefeld, Germany.

[2] Micron Oxford, Department of Biochemistry, University of Oxford, Oxford, UK.

Correspondence should be addressed to Thomas Huser (thomas.huser@physik.uni-bielefeld.de)

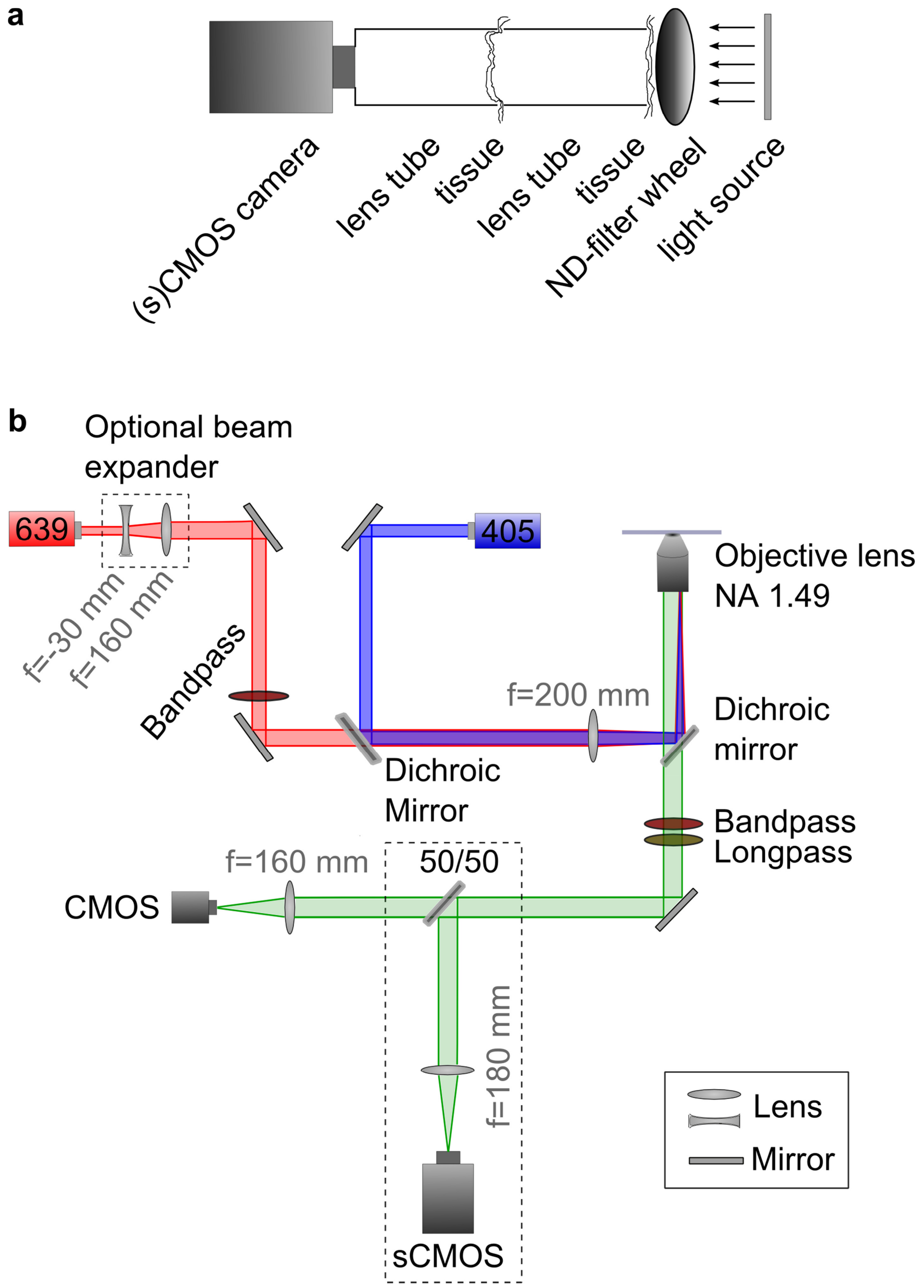

Supplementary Figure 1. Setups. **a**, Camera characterization is performed by adjusting the light intensity with a neutral density filter wheel and diffusing the beam by two layers of paper tissue. **b,** Microscope. The beam of a 639 nm laser is optionally extended by a telescope and combined with the beam of a 405 nm laser. These beams are jointly focused to the back-focal-plane of the objective lens to achieve TIRF illumination. The fluorescence signal is either equally split to the two different cameras (**Figure 2**) or by removal of the 50/50 beamsplitter directed to the CMOS camera only (**Figure 3**).

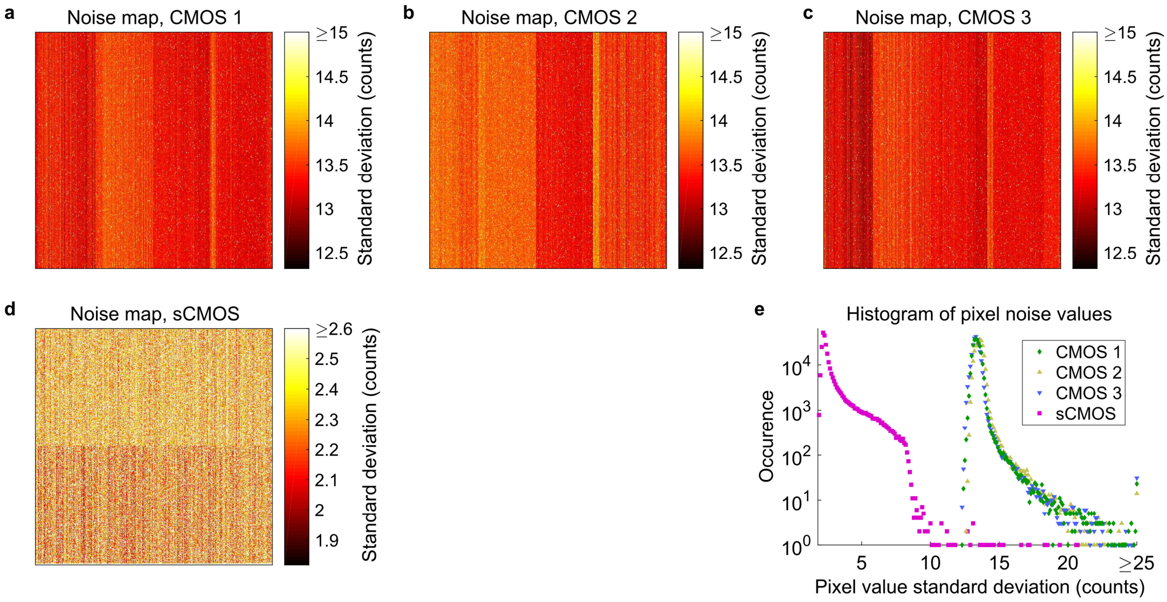

Supplementary Figure 2. Noise maps (**a-d**) and corresponding histograms (**e**) for all four cameras considered in this work. The three tested CMOS cameras possess similar distributions for the pixel noise values while the sCMOS camera shows remarkably lower noise.

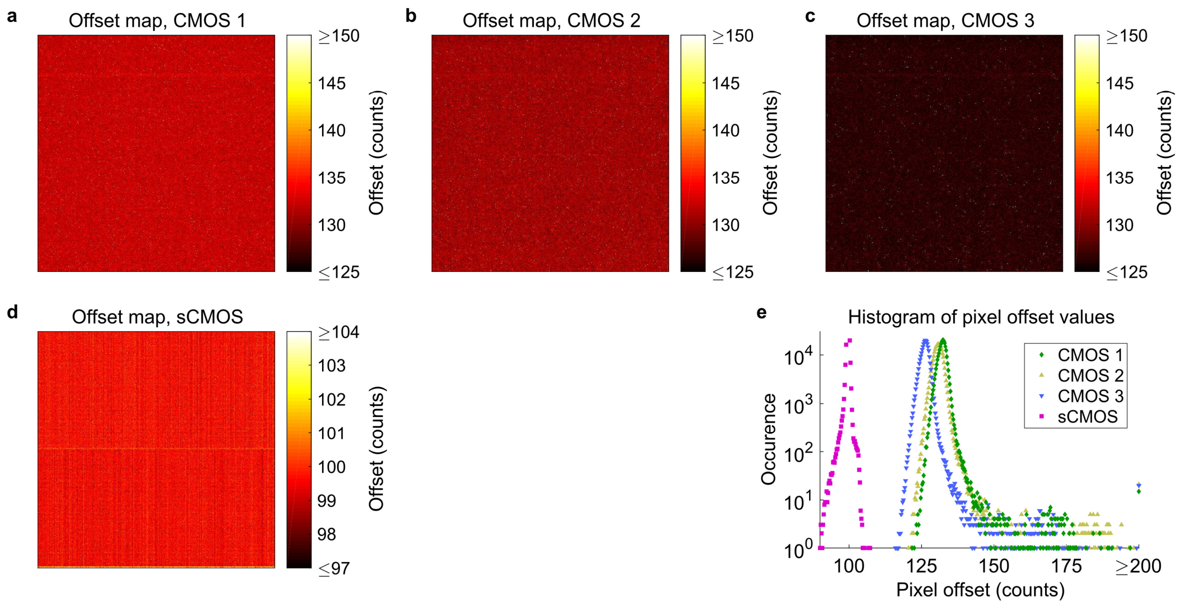

Supplementary Figure 3. Offset maps (**a-d**) and corresponding histograms (**e**) for all four cameras considered in this work. No obvious pattern is visible in the offset maps of the three CMOS cameras and the width of their distributions is about the same but significantly smaller for the sCMOS camera.

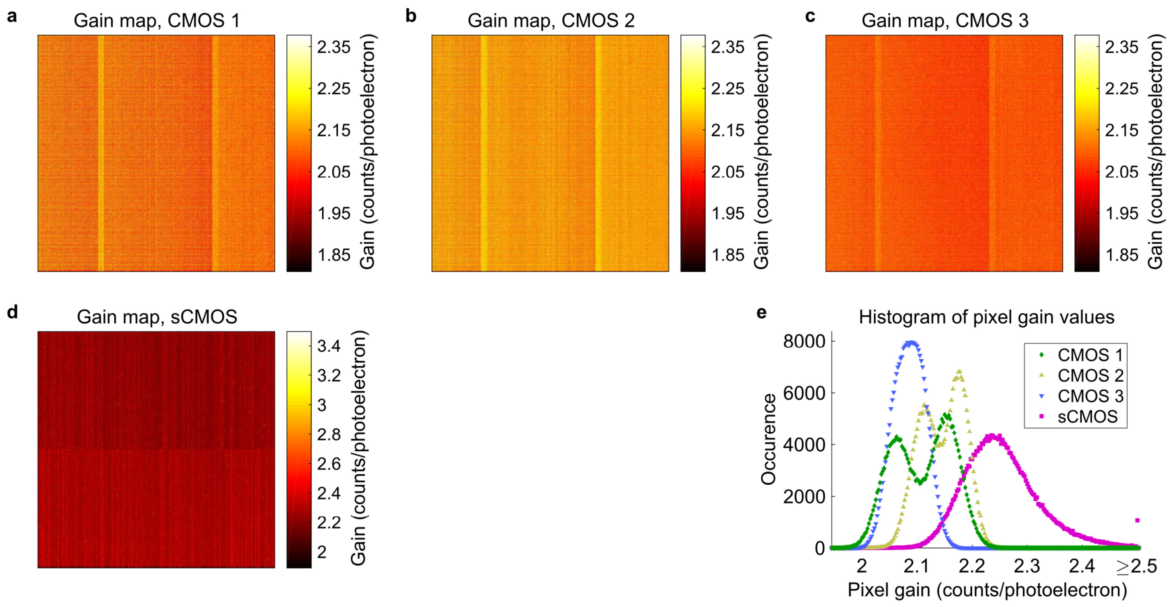

Supplementary Figure 4. Gain maps (**a-d**) and corresponding histograms (**e**) for all four cameras considered in this work. The pixel gain distributions for two CMOS cameras show two peaks while only one peak and a narrower distribution occurs for the third camera.

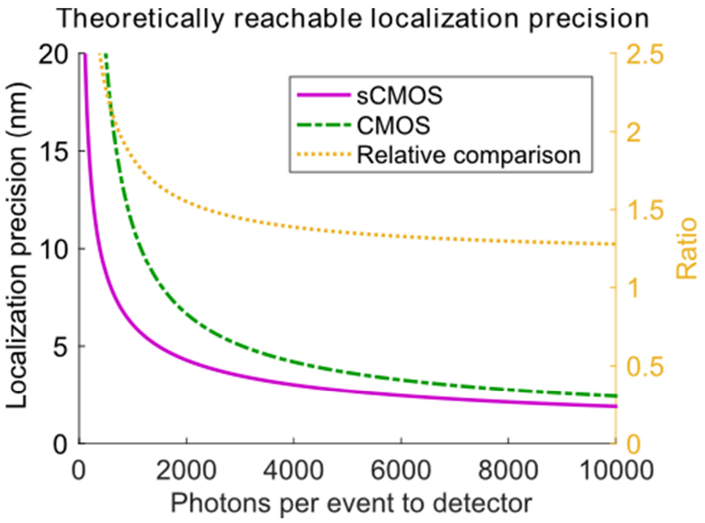

Supplementary Figure 5. Theoretically reachable localization precision for the two different cameras. Using the model of Mortensen et al.[1], we have calculated the localization precision under the assumption of

- noise only being induced by the cameras but no background noise from the imaging process,
- the number of detected photons being determined by the camera quantum efficiencies of 69 % in case of the sCMOS and 48 % in case of the CMOS camera, and
- a point spread function full-width-at-half-maximum corresponding to Rayleigh's criterion at an emission wavelength of 660 nm and a numerical aperture of 1.49.

The yellow curve shows the ratio between the localization precisions for the CMOS and the sCMOS camera as a function of the signal level. The ratio is significantly increased for low photon numbers due to the higher noise of the CMOS camera.

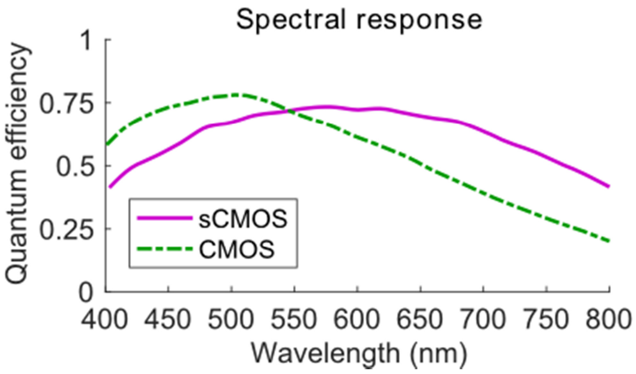

Supplementary Figure 6. Spectral response curves of the cameras used in this work. The curves were extracted from the camera data sheets[2,3] using the WebPlotDigitizer tool[4].

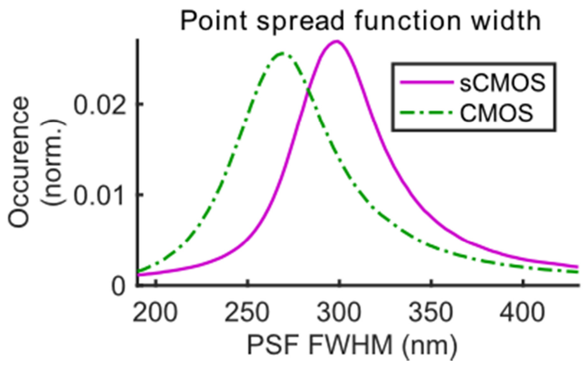

Supplementary Figure 7. Distribution of the point spread function (PSF) widths of the individual emitter detections for the experiment shown in **Figure 2b,c**. The theoretically expected value of the full-width-at-half-maximum (FWHM) is close to Rayleigh's criterion of $1.22\frac{\lambda}{2\mathrm{NA}} \approx 1.22\frac{660\ \mathrm{nm}}{2\cdot 1.49} \approx 270\ \mathrm{nm}$. This is almost reached by the modal value of 270.8 nm for the CMOS camera, but the modal value of 299.1 nm for the sCMOS camera is about 11 % higher. We first suspected this being caused by aberrations in the beam reflected from the 50/50 beamsplitter. Hence, we have tested the sCMOS camera in both the reflection as well the transmission paths behind the beamsplitter and also with four different tube lenses (two times f = 160 mm and two times f = 180 mm, all from Qioptiq). The modal value of the PSF FWHM was close to 300 nm for all tested configurations. We therefore conclude that the wider PSF is not induced by our setup but a property of our sCMOS camera itself. The difference in the PSF widths also explains that the curve of the localization precision model (**Figure 2c**) for the CMOS camera falls below the curve for the sCMOS camera for more than about 3,200 photoelectrons as we have used the experimentally determined values for the PSF width and noise to generate these curves. In contrast, optimal conditions were assumed for the curves shown in **Supplementary Figure 5** where the localization precision is better for the sCMOS camera for all photon numbers.

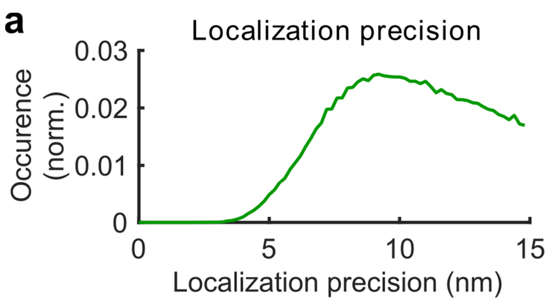

894 fps
Modal value localization precision: 9.2 nm

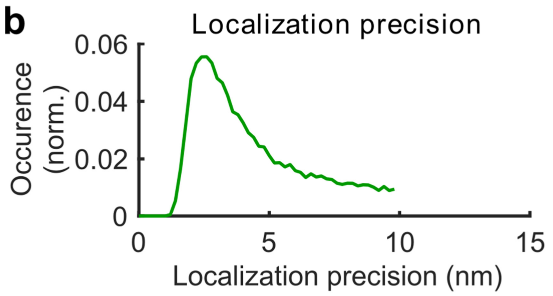

40 fps
Modal value localization precision: 2.6 nm

Supplementary Figure 8. Distribution of the localization precision for the *d*STORM images shown in **Figure 3b** (**a**) and **Figure 3c** (**b**).

Supplementary Note 1.

A homogeneous illumination of the camera detector is crucial if the mean and variance values are determined from single frames over all pixels of the detector. This might be a valid approach if a sufficient detector homogeneity can be assumed, e.g. in case of EM-CCDs. However, as (s)CMOS detectors feature significant pixel-to-pixel variations, this would lead to an overestimation of the true variance caused by photon shot noise. Hence, the mean and variance values are determined for each pixel separately from a time trace over several thousand frames (**Methods**) which results in the maps shown in **Figure 1** as well as **Supplementary Figure 2**, **Supplementary Figure 3** and **Supplementary Figure 4**.

As the computations are performed for each pixel independently, it is not necessary to achieve spatially homogeneous illumination of the detector. In contrast, care must be taken to achieve temporally homogeneous illumination intensity. If the light intensity varied over time, this would again lead to an overestimation of the variance. It has emerged that the emitted light power of different battery-powered flash lights decreases over time. Therefore, we used the TN-LED screen of a laptop with the charger plugged in as the light source in an otherwise dark room. The intensity between different measurements was varied using a neutral-density filter wheel and manually adjusted to achieve roughly equal spacing between the measurement points in terms of the mean signal (**Supplementary Figure 1a**).

Though spatially homogeneous illumination is not crucial, two layers of paper tissue were mounted at different distances with a lens tube before the camera chip. This resulted in almost uniform illumination to ensure that points in the variance over mean curve were measured at comparable signal levels for all pixels. However, as the gain is determined from the slope of a linear fit to this proportional relation, the exact positions of the measurement points along this curve are not important. While the precision of the single points along this curve depends on the number of frames for temporally constant illumination that are used to determine variance and mean, the precision of the fit to determine the gain depends on the number of the measured points. To ensure that both measurements are made with high precision, several thousand frames (4,000 to 8,192) were used for calculation of each point in the variance over mean curve and multiple points (15 to 20) were used for the fit of the linear relation, while in principle two points are sufficient. No significant variation in the quality of the fit was observed from the variations in the number of frames or number of points, respectively. Anyway, to not unnecessarily lower the achievable precision, all available measurements have been used for the computations in each case though the number of frames and data points varied between characterizations of the different cameras.

References

1. Mortensen, K. I., Churchman, L. S., Spudich, J. A. & Flyvbjerg, H. Optimized localization analysis for single-molecule tracking and super-resolution microscopy. *Nat Methods* **7**, 377-381, doi:10.1038/nmeth.1447 (2010).
2. Hamamatsu. Orca Flash 4.0 Data Sheet.
3. IDS. μeye UI-3060CP-M-GL Rev.2 Data Sheet.
4. Rohatgi, A. WebPlotDigitizer, http://arohatgi.info/WebPlotDigitizer. (2017).
